# Accelerated evolution of the mitochondrial genome in an alloplasmic line of durum wheat

**DOI:** 10.1186/1471-2164-15-67

**Published:** 2014-01-25

**Authors:** Andrzej K Noyszewski, Farhad Ghavami, Loai M Alnemer, Ali Soltani, Yong Q Gu, Naxin Huo, Steven Meinhardt, Penny MA Kianian, Shahryar F Kianian

**Affiliations:** Department of Plant Sciences, North Dakota State University, Fargo, ND 58108 USA; Department of Plant Pathology, University of Minnesota, St. Paul, MN 55108 USA; Computer Information Systems Department, The University of Jordan, Amman, Jordan; USDA-ARS, Western Regional Research Center, Albany, CA 94710 USA; Department of Plant Pathology, North Dakota State University, Fargo, ND 58108 USA; Cereal Disease Laboratory, USDA-ARS, Minneapolis, MN 55108 USA

**Keywords:** *Aegilops longissima*, *Triticum turgidum*, Heteroplasmy, Paternal leakage, *atp6*, Next generation sequencing, Cytoplasmic male sterility, Alloplasmic line

## Abstract

**Background:**

Wheat is an excellent plant species for nuclear mitochondrial interaction studies due to availability of large collection of alloplasmic lines. These lines exhibit different vegetative and physiological properties than their parents. To investigate the level of sequence changes introduced into the mitochondrial genome under the alloplasmic condition, three mitochondrial genomes of the *Triticum-Aegilops* species were sequenced: 1) durum alloplasmic line with the *Ae. longissima* cytoplasm that carries the *T. turgidum* nucleus designated as (lo) durum, 2) the cytoplasmic donor line, and 3) the nuclear donor line.

**Results:**

The mitochondrial genome of the *T. turgidum* was 451,678 bp in length with high structural and nucleotide identity to the previously characterized *T. aestivum* genome. The assembled mitochondrial genome of the (lo) durum and the *Ae. longissima* were 431,959 bp and 399,005 bp in size, respectively. The high sequence coverage for all three genomes allowed analysis of heteroplasmy within each genome. The mitochondrial genome structure in the alloplasmic line was genetically distant from both maternal and paternal genomes. The alloplasmic durum and the *Ae. longissima* carry the same versions of *atp6*, *nad6*, *rps19*-p, *cob and cox2* exon 2 which are different from the *T. turgidum* parent. Evidence of paternal leakage was also observed by analyzing *nad9* and o*rf359* among all three lines. Nucleotide search identified a number of open reading frames, of which 27 were specific to the (lo) durum line.

**Conclusions:**

Several heteroplasmic regions were observed within genes and intergenic regions of the mitochondrial genomes of all three lines. The number of rearrangements and nucleotide changes in the mitochondrial genome of the alloplasmic line that have occurred in less than half a century was significant considering the high sequence conservation between the *T. turgidum* and the *T. aestivum* that diverged from each other 10,000 years ago*.* We showed that the changes in genes were not limited to paternal leakage but were sufficiently significant to suggest that other mechanisms, such as recombination and mutation, were responsible. The newly formed ORFs, differences in gene sequences and copy numbers, heteroplasmy, and substoichiometric changes show the potential of the alloplasmic condition to accelerate evolution towards forming new mitochondrial genomes.

**Electronic supplementary material:**

The online version of this article (doi:10.1186/1471-2164-15-67) contains supplementary material, which is available to authorized users.

## Background

Mitochondria are a crucial component of every eukaryotic cell and provide indispensable functions including i) cell energy supply ii) synthesis of essential molecules such as phospholipids and heme, and iii) mediating multiple cellular signaling pathways including stress response, apoptosis and aging [1, 2]. It is widely accepted that mitochondria and chloroplast descended from free-living bacterial ancestors [3] that were acquisitioned into eukaryotic cells. Following their acquisition, most of the genes in these organelles were either lost or transferred to the nucleus [3]. Although these organelles possess their own genomes, most of their proteins (93-99%) are encoded in the nucleus, synthesized in the cytoplasm and then imported for use [4].

There is great variability in size and number of genes of eukaryotic mitochondrial genomes. The average size of a mitochondrial genome is about 15–60 kb in animals while in seed plants they range from 222 to 2,900 kb [5, 6]. The number of genes in the mitochondrial genome of eukaryotes is also variable, from 5 in plasmodium to 100 in jakobid flagellates and with average between 40 and 50 [6]. Recent sequencing of the hexaploid wheat (*T. aestivum* cv. Chinese Spring) mitochondrial genome revealed interesting features of this genome relative to other monocots such as rice and maize [7]. The size of this genome is 452 kb [7] compared to 491 kb for rice [8], and 536 to 740 kb for various *Zea mays* genotypes [9]. This mitochondrial genome of hexaploid wheat includes 55 known genes and 179 ORFs larger than 300 bp in size. About 15% of this genome is repetitive sequences, 3% is of chloroplast origin and 0.2% is of retro-element origin. The gene order of the wheat mitochondrial genome showed little synteny to that of rice and maize [7].

A diverse range of methods have been used for sequencing the whole mitochondrial genome of over fifty different species currently deposited at NCBI Organelle Genome Resources. At the beginning BAC library construction combined with Sanger sequencing was the only method for mitochondrial DNA (mtDNA) sequencing [7, 10–12], although currently next generation sequencing (NGS) is being used as a replacement [13–15]. The advent of NGS techniques (such as Roche/454 GS-FLX and Illumina Hiseq) set the stage for high throughput, inexpensive mitochondrial genome comparisons among diverse species [13]. However, the multipartite nature along with large and short repeated sequences [16] has made *de novo* assembly of the mitochondrial genome a challenge. Despite the challenges in *de novo* assembly the depth of sequencing provided by NGS is so high that is quite suitable for detection of mitochondrial genome variants in a cell [17].

Usually more than one type of mitochondrial genome (mitotype) occurs in a cell, defined as heteroplasmy. The ratio of different mitotypes in a heteroplasmic population may be variable, with one major mitotype dominant and others present in a very low proportion [18, 19]. Heteroplasmy seems to be an evolutionary strategy of maternally inherited plant mitochondrial genomes to compensate for lack of sexual recombination. Therefore different mitotypes in the cell accumulate as a reservoir of genetic variability and may undergo accelerated evolution [20]. Occasionally, some mitotypes are selected and amplified in the process called substoichiometric shifting, and become the prominent mitochondrial genome [20]. Prevalence of each mitotype is believed to be controlled strictly by nuclear gene(s) in a tissue specific manner [21]. An example of this interaction is the nuclear *Fertility restorer* (*Fr*) gene in common bean which decreases the male sterility associated mitotype (*pvs*) to a substoichiometric level that recovers the fertility [21]. A detailed study on the complex nature of the plant mitochondrial genome was conducted by analyzing the composition of three different genes, *atp4*, *atp6* and *rps7*, in alloplasmic and euplasmic lines of wheat [22]. This study suggested that paternal mitochondrial types are increased by up to 30% in alloplasmic lines but they tend to be silenced.

Alloplasmic lines are created when the cytoplasm of one species is replaced by that of another species through substitution backcrossing. This alloplasmic condition disrupts the interactions between the nucleus and cytoplasm which have co-evolved together as illustrated in alloplasmic lines of *Tigriopus* and *Drosophila*[23]. The disruption of nuclear mitochondrial (NM) interactions in plants by alloplasmic condition usually leads to cytoplasmic male sterility (CMS) [24, 25]. It is believed that pollen-forming tissue demand tremendous amount of energy and have a lower threshold for respiratory deficiencies than other plant tissue [26, 27]. Therefore in most cases the alloplasmic lines are CMS as well. In all cases examined, the CMS phenotype results from the presence of novel proteins in the mitochondria affecting mitochondrial function and pollen development [4, 28]. The new ORFs, leading to the production of novel proteins, are the consequence of highly rearranged mitochondrial genome [9]. Research also indicates alterations in the mitochondrial gene expression patterns in alloplasmic plants [29, 30].

Wheat has the largest collection of alloplasmic lines created in any species [31, 32], providing an un-precedent opportunity to analyze NM interactions. These lines have exhibited vegetative and physiological phenotypes different from their parents. Some alloplasmic wheat, with the *Ae. mutica* or the *Ae. crassa* cytoplasms, showed higher yield, increased tolerance to abiotic stresses, and improved seed quality [32–34]. In this study, the mitochondrial genomes of three *Triticum-Aegilops* species were sequenced and described; durum alloplasmic line (lo) durum with the *Ae. longissima* cytoplasm with the nucleus from the *T. turgidum* var*. durum* plus its two euplasmic parents. The (lo) durum line was developed and described by S.S. Maan [32]. The main focus of this study was to investigate the level of changes that occurred in the mitochondrial genome in the alloplasmic condition. Sequencing of mitochondrial genome in these three lines allows for analysis of changes in the mitochondrial genome of the alloplasmic line compared to its euplasmic parents.

## Results

### Genome assembly

The average sequence read length was 449 bp as expected for 454 GS FLX sequencing technology. The purification method [35] provided mitochondrial DNA free from nuclear DNA contamination which was confirmed by quantitative real time PCR. Based on sequencing read counts, 3 to 9% of overall reads were chloroplastic DNA and no nuclear DNA was detected. The majority of sequencing reads (66-79% of total reads) were used for mitochondrial genome assembly, with the exception of the *Ae. longissima* where only 32% of the reads were used. The remaining 12-28% of reads that were not assembled created additional contigs which contained valid mitochondrial sequences. These contigs may provide additional information about possible mitotypes present in smaller proportion relative to the most prevailing type presented in the final assemblies.

The mitochondrial genomes obtained for *T. turgidum* was 451,678 bp and sequence was similar to the previously sequenced *T. aestivum* cv. Chinese Spring [7]. Although sequences were nearly identical, 40 single nucleotide polymorphisms (SNPs) were observed between the two genomes, five were in known mitochondrial genes: *rps1*, *rps2*, *cox3* and *ccmFN*. The estimated size of the (lo) durum and the *Ae. longissima* genomes were 431,959 bp and 399,005 bp, respectively. Overall coverage of the sequenced genomes was 61 – 133x (Table 1), which allowed analysis of heteroplasmy.

**Table 1:** **Final assembly results for mitochondrial genomes of various** ***Triticum*** **/** ***Aegilops*** **species**

Species	Coverage (x)	Genome size (bp)	Ave. read length (bp)	# of contigs
*T. turgidum*	114	451,678	450	1
*Ae. longissima*	61	399,005	442	2
(lo) durum	133	431,959	436	1

### Genome structure

**Figure 1:**
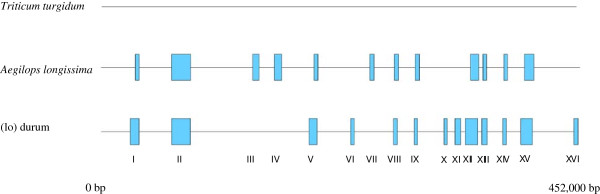
**Comparison of mitochondrial genomes in the alloplasmic lines and its parental lines based on reference assembly.** Blue boxes represent gaps (>3,000 bp) in assembly as compared with the *Triticum aestivum* (NC_007579.1) reference. The *T. turgidum* assembly has only one gap of 1,200 bp that is not indicated.

Using the *T. aestivum* cv. Chinese Spring as the reference, the *T. turgidum*, the (lo) durum, and the *Ae. longissima* mitochondrial genomes were assembled. The *T. turgidum* mitochondrial genome sequence is missing a region of about 1,200 bp (corresponding to 179,037 to 180,129 bp position) present in the *T. aestivum* mitochondrial genome (Figure 1). There were 12 and 13 regions with no reads for the *Ae. longissima* and the (lo) durum, respectively (Figure 1). However, nine of these regions (regions I, II, V, VII, VIII, IX, XII, XIII, XIV and XV) appeared to be in common, but there are size differences between some of them (especially at regions I, V and IX). Regions VI, X, XI and XVI were present in the *Ae. longissima* but not in the alloplasmic line. Regions III, IV, and VII were present in the alloplasmic line but not the *Ae. longissima* (Figure 1). These differences could indicate the presence of genome rearrangements, new genetic information or high polymorphism hindering complete assembly. These results indicate changes in the predominant genome and also the lack of these regions in the minor mitotypes.

**Figure 2:**
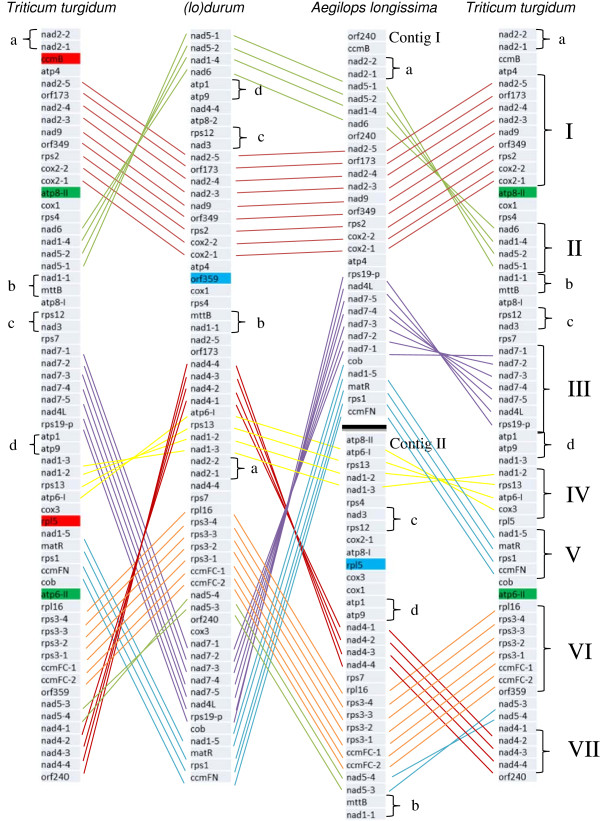
**Graphical representation of the synteny observed in the** ***Triticum turgidum,*** **the (lo) durum and the** ***Aegilops longissima*** **mitochondrial genomes based on gene localization.** Pairs of genes found together in all lines a, b, c, d and gene blocks (I -VII) are shown. Black line in the *Ae. longissima* indicates the position of border between the two contigs that could not be merged by *de novo* assembly. Genes labeled as green are present only in one copy in the (lo) durum and the *Ae. longissima*, blue are missing from either the *Ae. longissima* or the (lo) durum and gene labeled in red are not present in our final assembly of the (lo) durum line.

Considering the gaps in the reference assembly, *de novo* assembly of the genomes could reveal structural changes reflecting recombination and/or other rearrangement events within the mitochondrial genomes. Within these regions are blocks of DNA that share high sequence similarity (about 99%), indicating conservation of mitochondrial genome content, but not genome structure. To precisely describe the possible recombination events within genomes and the differences which appear in the (lo) durum line as an effect of the alloplasmic condition, a detailed gene order description was initiated. Based on the syntenic relationship between genomes (Figure 2), three gene categories were established. 1) genes, which do not follow any recognizable pattern, 2) gene pairs that move together and, 3) genes that tend to remain together in blocks. Examination of these blocks revealed that there were 19 recombination/rearrangement events between the *T. turgidum* and the alloplasmic (lo) durum and 13 between the *Ae. longissima* and the alloplasmic (lo) durum. Four pairs of genes (labeled a, b, c and d) and seven conserved gene blocks can be detected between the alloplasmic line and both parents (Figure 2). These blocks account for 44 genes (or gene exons) out of 61 total, providing evidence for the relative stability of the gene order between these species. Three gene blocks show rearrangements within them: blocks I, V and VI, with lengths of 28,642 bp, 20,955 bp and 22,939 bp in the *T. turgidum* (Figure 2). Blocks I and V conserve the same gene order except for *atp4* (Block I) and *cob* (Block V) having moved from one end of the block to the other end. In block VI, *orf359* located after *ccmFC* exon-2 in the *T. turgidum* and *nad5* exons 3 and 4 are reversed relative to (lo) durum. The location of *rps7* has been moved adjacent to block VI in the *Ae. longissima* and (lo) durum. The gene blocks consist of about 32% (143,445 bp) of the whole genome.

### Major differences in genes

The 39 protein coding genes, three rRNA and 17 tRNA genes characterized in the *T. aestivum* mitochondrial genome [7] were identified in the sequenced lines. Majority of the genes are highly conserved between mitochondrial genomes in these three lines (Additional file 1: Table S1).

**Figure 3:**
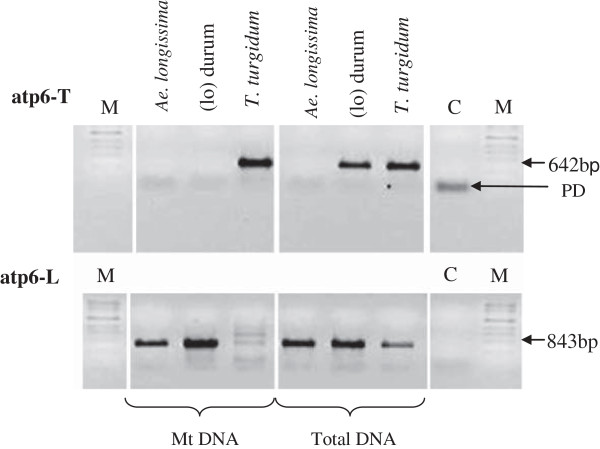
**Differentiation of the** ***atp*** **6 alleles revealed by amplification using two sets of forward primers designed based on mitochondrial genome sequences (Additional file** **3** **: Table S3).** The atp6-T amplification of *atp*6 allele from the *T. turgidum* and atp6-L amplification of *atp*6 from the *Aegilops longissima* and the (lo) durum line. DNA was separated on a 1% agarose gel. M indicates bp size marker, C indicates control PCR, PD - primers dimers.

Six genes (*atp6-*1, *rps19*-p*, nad9, nad6, cob* and *cox2* exon 2) had many nucleotide changes in the (lo) durum and the *Ae. longissima* genomes compared with the *T. turgidum* genome. One difference is recognized in the *atp6* gene between the *T. turgidum* (1161 bp) and the (lo) durum line (1248 bp). This mitochondrial gene, encodes ATP *synthase* F0 subunit 6. The *apt6-*1 gene found in the *Ae. longissima* and the (lo) durum (designates as *atp6*-L) has an altered pre-sequence in comparison to the *T. turgidum* (designated as *apt6-*T). However, there was no difference in the surrounding region of this gene which is conserved among all three species. In addition, they share a highly similar core region of 869 bp (Additional file 2: Figure S1). A BLAST search found the pre-sequence of *atp6* from the *Ae. longissima* and the (lo) durum is present only in the male sterile alloplasmic *T. aestivum* line with the *Ae. kotschyi* cytoplasm reported recently [36]. In addition to having the characteristic pre-sequence for the female parent, the (lo) durum has a number of SNP’s in the core region when compared to the *T. turgidum* gene. These changes are C/A^520^, A/T^543^, T/A^544^, A/T^580^, T/A^581^, A/C^595^, T/G^639^, including SNP T/G^560^ specific only to the (lo) durum. The amplification of *atp6*-L and *atp6*-T alleles based on mitochondrial DNA and total DNA isolation was performed to confirm the sequences obtained from the assembly (Figure 3). The *atp6*-T amplification was detected in the *T. turgidum* mtDNA*,* but not in the *Ae. longissima* and the (lo) durum mtDNA. When PCR was performed based on the total DNA, no product was observed in the case of the *Ae. longissima,* but for the (lo) durum, and the *T. turgidum* a proper allele was amplified. Amplification of *atp6-*L allele (mitochondria DNA only) confirmed its presence in the *Ae. longissima* and the (lo) durum. The *T. turgidum* showed no proper amplification in mtDNA; however, the allele seems to be present in its nucleus due to amplification in total DNA.

Another major gene difference was observed in *nad9* (Additional file 4: Figure S2). A four-nucleotide deletion (TGTG^134-137^) was present in *nad9* of the *Ae. longissima.* The alloplasmic durum line doesn’t share this deletion. However, a characteristic di-nucleotide difference, TG/CA^134-135^ in relation to the *T. turgidum*, was observed resulting in V/A^45^ substitution in the *nad9* protein. The four-nucleotide deletion found at position 135 creates a frame shift in the *Ae. longissima* gene and generates a STOP codon at position 158 (TAG^158-160^). This creates an alteration in protein length compared to the *T. turgidum*. Beside these changes, an additional three SNPs were observed in the region before the STOP codon, but no other polymorphisms were found across the rest of the gene. There is a SNP A/C^118^ specific to (lo) durum, and two SNPs C/A^122^ and A/T^125^ found in both the *Ae. longissima* and (lo) durum. Each of the SNPs creates amino-acid changes, N/H^40^, S/Y^41^, K/I^42^ respectively, in the *nad9* protein.

The *nad6* mitochondrial gene is responsible for coding subunit 6 of Complex I, the NADH-ubiquinone oxidoreductase, which is built from at least 30 different subunits that are mainly encoded by the nucleus. Multi-alignment of the *nad6* gene alleles (Additional file 5: Figure S3) shows that a truncated version of *nad6* is present in the *Ae. longissima* and the (lo) durum lines, but not in the *T. turgidum*. The conserved region begins at the ATG start codon and continues until nucleotide 703. No polymorphism was found between the alloplasmic line and its parents within the conserved region of the gene.

A nine-nucleotide deletion (AAAGGTTGG^122-130^) in the *rps19-*p pseudo gene was recognized in the *T. turgidum* mitochondrial in relation to the (lo) durum and the *Ae. longissima.* There are no allelic differences between the last two lines (Additional file 6: Figure S4). A polymorphism was also identified in the sequence of *cob* and *cox2* exon-2 when the *T. turgidum* was compared to the (lo) durum and the *Ae. longissima*. Both genes were 10 bp shorter at the 3′end in the *T. turgidum* compared to the other two lines (data not shown).

### Polymorphism of genes within and between chondriomes

There were multiple alleles of a gene present at different stoichiometric levels due to heteroplasmy in chondriome of each line. To identify nucleotide changes in the genes between chondriomes, the predominant allele for each line was used for comparison. In total 10 and 14 SNPs were identified for the *Ae. longissima* and the alloplasmic line compared to the *T. turgidum*, respectively (Tables 2 and 3). The total number of nucleotide variations observed in the alloplasmic (lo) durum line was higher than any of the other species examined. Six SNPs found in *cox3* exon-2 (GA/TC^687^), *mttB* (T/G^41^), *rps2* (A/T^233^), *rps4* (C/A^495^), and *rps13* (A/C^170^) were identified as unique to the alloplasmic line, and differentiate it from both parents. Our data shows that the ribosomal coding genes are the most variable when compared to the other mitochondrial protein coding genes. Three out of six SNPs observed between the (lo) durum and the *Ae. longissima* were found in the ribosomal protein coding genes *rps2*, *rps4* and *rps13* (Table 3).

**Table 2:** **Nucleotide variation(s) in ribosomal protein coding genes**

		***T. turgidum***	***Ae. longissima***	(lo) durum	Amino acid change
**Ribosomal proteins**	rps1-1*	+	−	−	−
	*rps1-2*	−	C/A^33^, C/T^397^	C/A^33^, C/T^397^	No change, T/I
	*rps2-1*	+	+	−	Y/F
	*rps2-2*	−	−	**A/T** ^**233**^	
	*rps4-1*	+	−	−	−
	*rps4-2*	−	G/T^146^, T/G^236^	−	R/L, L/R
	*rps4-3*	−	−	G/T^146^, T/G ^236^, **C/A** ^**495**^	R/L, L/R + no change
	*rps13-1*	+	−	−	−
	*rps13-2*	−	A/C^45^	−	No change
	*rps13-3*	−	−	A/C^45^, **A/C** ^**170**^	No change, E/A
	*rpl5-1*	+	−	−	−
	*rpl5-2*	−	G/T^28^	G/T^28^	
**tRNA**	*His tRNA-1*	+	−	−	−
	*His tRNA-2*	−	C/T^10^, T/C^22^	C/T^10^,T/C^22^	Not applicable
**Variations** ^**&**^		0	6	9	

Arabic numbers indicate the SNP position relative to the start codon.

+/− Indicates the presence/absence of gene allele in a particular genome.

*Gene with multiple alleles. Reported here are the most abundant alleles.

^&^Summary of nucleotide variation observe only in genes with one known allele of that gene within species.

Listed are the nucleotide changes found in the (lo) durum, and the *Ae. longissima* with the *T. turgidum* as the reference sequence*.* The SNPs characteristic for the alloplasmic line are bold.

**Table 3:** **Nucleotide variation(s) within the coding genes of the electron transport complexes**

	Gene	***T. turgidum***	***Ae. longissima***	(lo) durum	Amino acid change
**Complex I**	nad3-1*	+	−	−	−
	*nad3-2*	−	T/C^185^	T/C^185^	L/P
**Complex IV**	*cox3-1*	+	−	−	−
	*cox3-2*	−	A/C^157^, **GA/TC** ^**687**^	−	I/L, QM/HL
	*cox3-3*	−	−	A/C^157^	I/L
**Complex V**	*atp1-1*	+	−	−	−
	*atp1-2*	−	A/T^1431^	A/T^1431^	No change
	atp8-I-1*	+	−	−	−
	*atp8-I-2*	−	C/A^239^	−	
	*atp8-I-3*	−	−	C/T^39^, A/G^149^, G/A^166^	
	atp8-II-1*	+	−	−	−
	*atp8-II-2*	−	C/A^239^, G/A^463^, T/C^467^	−	
	*atp8-II-3*	−	−	G/A^462^, T/C^467^	
**Other proteins**	*mttB-1*	+	+	−	−
	*mttB-2*	−	−	**T/G** ^**41**^	L/W
**Variations** ^**&**^		0	4	3	

Arabic numbers indicate the SNP position relative to the start codon.

+/− Indicates the presence/absence of gene allele in a particular genome.

*Gene with multiple alleles. Reported here are only the most abundant alleles.

^&^Summary of nucleotide variation observe only in genes with one know allele of that gene within species.

Listed are the nucleotide changes found in the (lo) durum, and the *Ae. longissima* with the *T. turgidum* as the reference sequence*.* The SNP’s characteristic for each line are bold.

**Figure 4:**
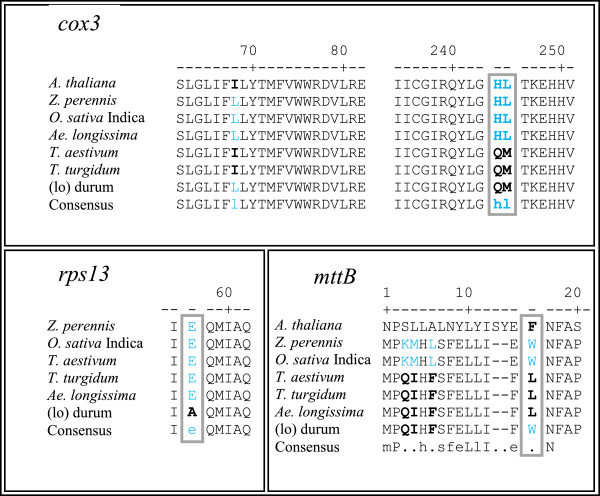
**Multi-alignment of protein sequences related to** ***cox3, rps13, mttB*** **genes from different species.** Gray boxes indicate amino acid changes which are functionally important. Only fragment of genes are shown where polymorphism of interest was present.

Differences in nucleotide sequences between genomes results in changes in amino acid sequences and potentially function of proteins. Several of the observed differences (Table 3) also change the amino acid sequences. To try to predict whether the observed amino acid changes would result in altered activities, the amino acid sequences of the *cox3*, *rps13*, and *mttB* proteins were compared to other related monocots and *Arabidopsis thaliana*. All of the observed changes were found in other species with the exception of the A/E^57^ change in the *rps13* gene of the (lo) durum which was unique to that line (Figure 4).

### Variations in copy number of genes

Some genes such as *ccmB* and *rpl5* (Figure 2) were missing in the (lo) durum mitochondrial genome. The *rpl5* gene is present in the *Ae. longissima* genome. There were also differences in the number of gene copies for instance *atp6* and *atp8* were present in two copies (alleles) in the assembled *Ae. longissima* mitochondrial genome but just one copy in the assemble (lo) durum genome. Three copies of *rrn18* were identified in *T. turgidum* and in the (lo) durum genomes, while just one copy was present in the *Ae. longissima*. No polymorphism within these genes was identified among the species. Two copies of the *rrn26* gene were identified in the *T. turgidum,* two copies in the *Ae. longissima* (with one copy having the T/C^743^SNP), and one copy in the (lo) durum.

### SNPs between and within the chondriome of each line

**Figure 5:**
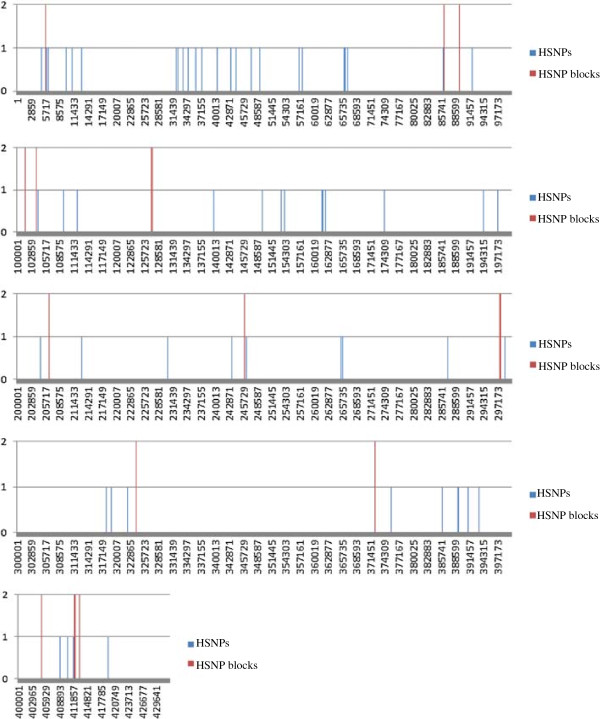
**HSNP distribution across the (lo) durum mitochondrial genome.** Blue lines indicates HSNP positions, red line indicate the position of HSNP blocks. We found 216 HSNPs in total, out of them 63 were HSNPs and 155 created 15 HSNP blocks. The overall HSNP density is 1HSNP/7,091 bp.

The existence of heteroplasmy was confirmed based on the existence of SNPs within chondriome of each line studied hereafter referred to as heteroplasmic SNPs (HSNPs). Within the chondriomes of the *T. turgidum*, the (lo) durum, and the *Ae. longissima,* 244, 218, and 344 HSNPs were identified, respectively (Additional file 7: Table S2). During the SNP search multiple regions of higher HSNP density (HSNP blocks) were evident compared to regions with only a single nucleotide change. These regions were unevenly distributed throughout the mitochondrial genomes. In the *T. turgidum* 22 HSNP blocks were identified containing 151 HSNPs, in the (lo) durum 15 blocks with 155 HSNPs (Figure 5) and in the *Ae. longissima* 14 regions, with 99 HSNPs in contig I and 13 regions, 147 HSNPs in contig II. No specific pattern of HSNP blocks was recognized. The number of HSNPs outside of these HSNP blocks were 93, 61, 98 in the *T. turgidum*, the (lo) durum and the *Ae. longissima* respectively (Additional file 7: Table S2).

A polymorphism search between the final consensus sequences of each genome was also performed. In total, 349 and 740 SNPs found comparing the (lo) durum line with the *Ae. longissima* and the *T. turgidum* respectively. There were also 731 SNPs between the *Ae. longissima* and the *T. turgidum.*

### ORF identification and comparison

Previously, several ORFs (designated as 359, 240, 173 and 349) have been identified in the *T. aestivum*[7]. The sequenced species were checked for the presence and potential polymorphism in these ORFs. The *orf173* and *orf240* did not show any polymorphism among sequenced species. The *orf349* in the alloplasmic line and the *Ae. longissima* carried a di-nucleotide difference of TT/AG^68^ when compared to the *T. turgidum* genome*.* The *orf359* from the *T. turgidum*, which was identical to the *T. aestivum*, was not present in *Ae. longissima*. Only a portion of that gene, from 73 bp to 1,080 bp was found in the (lo) durum. This ORF is significantly different in the alloplasmic line with 48 SNPs, three di-nucleotide changes, one tri-nucleotide change, and one triplet nucleotide insertion in regards to the *T. turgidum*’s copy of the ORF. This appears to be the most polymorphic region found in the sequenced genomes when compared to the *T. turgidum* (Additional file 8: Figure S5).

The *T. turgidum*, the (lo) durum, and the *Ae. longissima* genomes have 184 ORFs, 191 ORFs, and 163 ORFs with sizes larger than 300 bp, respectively. These ORFs consist of 81,978 bp (18.1%), 90,237 bp (20.8%) and 78,651 bp (19.7%) of the total genome size in each species, respectively. The alloplasmic line has the largest number of ORFs compared to its parents and the highest percentage of total ORF sequence per genome. To determine if there are species specific ORFs, a comparison of the ORFs was performed. There were 27 ORFs in the (lo) durum which are not present in the *T. turgidum* and the *Ae. longissima*, 15 ORFs present only in the *Ae. longissima*, and 26 ORFs only in the *T. turgidum*.

The ORFs unique to the alloplasmic line are further described in Table 4. Based on a Mega BLAST search against the NCBI database ORFs 0, 1, 3, 50, 61, 74, 97, 108, 159, 177 hit no similar sequences, possibly making them specific to this line. The ORFs 63, 65, 112 from the alloplasmic durum line are also present in the male sterile, alloplasmic line of *T. aestivum*[36] with 99% or more similarity (Table 4). The ORFs 122 and 146 are fully present in the alloplasmic *T. aestivum*[36]*,* but only a portion of these ORFs exist in normal common wheat. Additionally, in male fertile and male sterile lines of the *T. aestivum, orf14*, and *orf113* are different from that found in the alloplasmic durum. Based on its composition, *orf113* (396 bp) from the (lo) durum is composed of fragments of other known and functional mitochondrial genes *rps2*, *cox1*, *nad4* exon-2, and *rps19*-p (Additional file 9: Figure S6).

**Table 4:** **Mega BLAST results of unique ORFs for the (lo) durum line**

	Alloplasmic, CMS ***T. aestivum*** line (GU985444.1)		Normal, ***T. aestivum*** (EU534409)	
ORF#	Coverage (%)	Identity (%)	Coverage (%)	Identity (%)
14	14	100	14	97
26*	100	97	100	97
63	100	100	−	−
65	100	99	−	−
84	−	−	31	100
112	100	99	−	−
113	85	99	91	99
114	100	100	10	98
122*	100	97	100	98
138	22	100	−	−
146	100	98	66	98

*These two ORFs are not present in the final *T. turgidum* assembly.

− indicates absence of ORF in that line.

## Discussion

The chondriome is known to carry different types of mitochondrial genomes and showing polymorphism within each cell and tissue type [21]. Most reports on heteroplasmy in plants are based on sequencing of a few genes or ORFs [18, 22, 37]. The next generation sequencing method used in this study allowed deep sequencing with a range of 61-102x coverage in the mitochondrial genome of all three lines. This provided a clearer picture of heteroplasmy within each mtDNA (mtDNA hereafter will refer to different types of mitochondrial genomes or mitotypes presented in heteroplasmic condition in a plant). Heteroplasmy was observed in inter-genic space as well as genic space within each genome. Most mtDNA sequencing in wheat to date has been based on BAC or large cosmid library construction [7, 36]. This may make genome assembly easier due to the lower complexity of the data, but it also can limit the understanding of heteroplasmy and substoichiometric changes of the mitotypes in the chondriome.

This study provides for the first time a detailed analyses of an alloplasmic line of wheat in comparison to its two euplasmic nuclear and cytoplasmic donor lines. Analysis of the mitochondrial genome of an alloplasmic wheat with the *Ae. kotschyi* cytoplasm reported by Liu and his colleagues [36] lacked the sequence information of the genome from the euplasmic *Ae. kotschyi* maternal line for a better comparison. Reference assembly identified, several gaps in the mitochondrial genomes of the *T. turgidum*, the *Ae. longissima* and the (lo) durum as compared with that of the *T. aestivum* (Figure 1). Genome rearrangements and structural changes were evident using the *de novo* assembly of the mitochondrial genomes of the three lines (Figure 2). Mitochondrial genome of the alloplasmic line was not only distinct from the *T. turgidum* but also different from its maternal parent the *Ae. longissima*. Differences in gene order have not only been reported between different species of the same family, because of gene shuffling [7], but also among different ecotypes of a single species such as *Arabidopsis*[37]. Different genome structures have been observed comparing euplasmic lines with the CMS lines in maize [9], rice [13] and wheat [36]. Structural changes between the three genomes were expected, but the degree of these changes, which occurred in less than 50 years for the (lo) durum line, is quite significant. The rearrangements observed between the (lo) durum and the *Ae. longissima* mitochondrial genomes were greater than that observed for the *T. turgidum* and the *T. aestivum*, which are separated by 10,000 years of evolution [38]. The *Ae. longissima* mitochondrial genome has undergone a drastic structural change possibly as a result of introducing a new nuclear genome and production of a live alloplasmic line [39].

In alloplasmic conditions, incompatibility between the encoded gene products from nucleus and mitochondria could lead to altered mitochondrial function. The enzymes of the inner mitochondrial membrane which contain subunits encoded from both mitochondria and nuclear genomes may not be compatible and less functional in the newly formed alloplasmic line [40]. This pressure in addition to possible changes in recombination, due to improper function of nuclear gene(s) such as *MSH1* and *RECA3*[41] that control recombination in the mitochondrial genome, can lead to high frequency of heteroplasmy. The substoichiometric changes in favor of mitotypes that survive better in the new condition will accelerate evolution. This accelerated evolution of newly formed mitotypes can lead to multiple types different from the maternal mitochondrial genome in organization and genetic information, as found here and in other species [9, 13]. Therefore, in the alloplasmic CMS lines, not only a larger number of newly formed mitotypes exist, but also the stoichiometric shift changes the predominant mitotype from the maternal type to the one best suited to the new condition [20].

Multiple synteny blocks were observed between different lines possibly indicating sites of recombination (Figure 2). Studies on wheat mitochondrial gene expression indicated that *nad6* and *nad1* (exon d) are co-transcribed [42]. Both genes were found to be present in Block II, despite the high structural differences observed. Therefore, the co-existence of genes in the same block may be related to their functional association. At least 10 large repeated sequences exist in the wheat mitochondrial genome making it possible to have multiple sub-genomic circles in a cell. Multiple genome rearrangements, likely caused by recombination in mitochondrial repeated sequences, would result in the conserved coding regions often flanked by different sequences [40]. The co-existence of different mitotypes in a cell after these rearrangements may have hampered the production of a contiguous assembly of the whole wheat mitochondrial genome. Although reference assembly with the *T. aestivum* mitochondrial genome was possible for the *T. turgidum*, it was problematic for the other two lines. *De novo* assembly in these situations provided a better and more complete picture of the genome and its organization. The *de novo* assembly of mitochondrial genome was a challenge in our study due to the multipartite nature and rearrangements through recombination. The same difficulty was also faced by other groups using next generation sequencing of mitochondrial genome [13]. The *de novo* assembly using MIRA program followed by manual editing resulted in one contig of ~432 kb and two contigs with combined length of ~399 kb for the (lo) durum and the *Ae. longissima,* respectively. This emphasizes the necessity for developing more bioinformatics tools that are specific for mitochondrial genome analysis.

All previously characterized genes in the *T. aestivum* mitochondrial genome were present in the alloplasmic line and its parents. Despite conservation in gene content, multiple genes showed considerable sequence differences. The alloplasmic durum and the *Ae. longissima* line shared the same sequence variation in a number of genes such as *atp6-*1, *nad6*, *rps19*-p, *cob* and *cox2* exon-2 when compared to the *T. turgidum*. The *atp6* subunit of F_0_F_1_ – ATP synthase is considered to be a mitochondrial encoded gene. Early studies recognized the chimeric structure of *atp6* in *Triticum* species [43]. Later, it was found that the presence of those chimeric versions were associated with cytoplasmic male sterility in rice, where proper RNA editing of altered *apt6* could restore male fertility [44]. The same differences in *atp6-*1, *nad6*, *rps19*-p, *cob* and *cox2* exon 2 identified in this study have been reported by Liu et al. [36] in an alloplasmic line of *T. aestivum* with the cytoplasm of *Ae. kotschyi.* These appear to be the common differences between the *Triticum* and *Aegilops* genera rather than being due to the CMS condition as reported by Kawaura [22] for *atp6.*

To confirm the allelic differences of *atp6* between the (lo) durum and its parental lines, a complimentary PCR was performed with specific primers for each allele after genome sequencing (Figure 3). It seems the alloplasmic and its euplasmic maintainer have both *atp6-*T and *atp6-*L versions of the gene. However, *atp6-*T version appears to be only present in the (lo) durum nucleus rather than in mitochondria. The same situation seems valid for the presence of *atp6-*L in the *T. turgidum* nucleus. Recently it has been shown that two versions of *atp6* are present in the *Ae. crassa*, *T. aestivum* cv. Chinese Spring and the alloplasmic line of Chinese spring with the *Ae. crassa* cytoplasm [22]. The *atp6-*CR (*crassa*) version in Chinese Spring and *atp6-*AE (*aestivum*) version in the *Ae. crassa* were present in less than 10% of the mitochondrial pool in the cell. However, the frequency of *atp6-*AE was 30% in the alloplasmic line possibly due to paternal leakage. There was no obvious amplification for *atp6-*T in the (lo) durum or the *Ae. longissima* mtDNA and *atp6-*L in the *T. turgidum* mtDNA (Figure 3). Therefore, presence of a nuclear copy of both genes is likely.

Three different alleles of *nad9* were identified in this study. The allele found in the alloplasmic line is more similar to that of the paternal *T. turgidum* line than the *Ae. longissima*. This provides another evidence of paternal leakage during the creation of the alloplasmic line. Paternal leakage and its contribution to heteroplasmy has been indicated by numerous studies [19, 22, 45–47]. In wheat paternal leakage was investigated in detail in the alloplasmic hexaploid wheat having *Ae. crassa* cytoplasm [22]. It seems the proportion of paternal genes in the alloplasmic line increases by each backcrossing with the paternal line and then remains at a constant level [22]. The sequence of *nad9* in the (lo) durum has similarities to both parents. Therefore, this copy of *nad9* not only shows paternal leakage but also suggests that a recombination between the maternal and paternal mitochondrial genomes may have occurred. Since the *Ae. longissima* copy of the gene is absent in the (lo) durum, paternal leakage was either high for this gene and/or the nucleus of the *T. turgidum* selected in favor of the recombinant version which was similar to its own. It is known that nuclear genes determine the stoichiometry of alternative mitotypes [47]. Therefore, the second reason is most likely to be valid.

Two genes, *rpl5* and *ccmB* were missing in the final assembly of the (lo) durum. The *rpl5* gene encodes a ribosomal protein, responsible for rRNA maturation and formation of the 60S ribosomal subunits. Its function could be critical to the survival of the alloplasmic line. Both *rpl5* and *ccmB* genes were present in the raw assembly data but not included in the final assembly. In a recent study on sequencing the mitochondrial genome of a CMS line of *T. aestivum*, the lack of *rpl5* gene was associated with the CMS phenotype [36]. The lack of this gene in the final assemblies of the (lo) durum in our study and the (*Ae. kotschyi*) alloplasmic line [36] may be related to the proportion of mitochondrial DNA carrying that gene by means of substoichiometric shifting [48]. It is possible that the *rpl5* gene exists in a mitotype which is in a much smaller proportion compared to the major mitotype in the cell.

Besides major changes discussed, a nucleotide polymorphism search was performed within known mitochondrial genes in the sequenced species. Out of six SNPs observed between the (lo) durum and the *Ae. longissima*, three were in ribosomal protein coding genes *rps2*, *rps4* and *rps13.* Nucleotide variation within ribosomal genes was also observed in the alloplasmic line of *T. aestivum*, where among other, differences in *rps2*, and *rps4* were recognized [36]. This indicates that ribosomal genes are the possible “hot spots” for mutation in alloplasmic lines. Since ribosomal proteins are responsible for protein expression, these differences may be important to our understanding of CMS phenotype in plants. These findings suggest the possibility of creating potential SNP based markers to investigate other cytoplasm in alloplasmic lines of wheat. The functional effect of nucleotide variation in *cox3, rps13,* and *mttB* gene was evaluated in comparison to *A. thaliana, Zea perennis, T. aestivum* and *Oryza sativa* sub. Indica (Figure 4). Amino acid variations found in these genes are common among various plant species except for the change in *rps13* leading to an amino acid change unique to the alloplasmic line. This data supports the hypothesis of accelerated evolutionary changes in alloplasmic lines observed here and in another study [49].

Nucleotide polymorphism could be detected within each chondriome and categorized as single HSNPs and HSNP blocks. The HSNP blocks within each chondriome is an indicator of heteroplasmy in each genome and could possibly be used to investigate the proportion of particular mitotype in the genome. Overall mitochondrial genome polymorphism comparison between chondriome of each line showed an expected result that the alloplasmic line is closer to the *Ae. longissima* than to the *T. turgidum*. In the alloplasmic durum line, one particular region of DNA, designated as *orf359*[7], showed the highest level of polymorphism compared to other regions of the genome. Gene content and order showed that *orf359* does not exist in the cytoplasm donor line, but was completely conserved (sequence and position) among the *T. aestivum* and the *T. turgidum* genomes*.* Existence of *orf359* in the alloplasmic durum line is additional evidence for paternal leakage, although it was highly mutated. Since the (lo) durum line has been developed through a complicated backcrossing scheme including the (lo) *T. aestivum cv.* Selkirk and the *T. timopheevii*[39], the impact of these changes may have influenced the new ORF composition. Sequencing of the *T. timopheevii* mitochondrial genome can provide a better insight to these observations.

Several open reading frames specific to the alloplasmic line were detected, implying that alloplasmic condition can lead to creation of new ORFs. These new ORFs can contribute to CMS and other characteristic phenotypes in the (lo) durum line. Interestingly, ORFs 63, 65, 112 from the alloplasmic durum line were also observed in the alloplasmic hexaploid wheat containing the *Ae. kotchyi* cytoplasm [36]. The occurrence of these ORFs might be related to the alloplasmic condition for they were not present in the maternal lines. As both alloplasmic lines having these ORFs are CMS, it is possible that their existence are associated with the CMS condition. The most characteristic ORF recognized in this study was chimeric *orf113* (Additional file 9: Figure S6) composed entirely of four fragments of other mitochondrial genes, including *rps2*, *cox1*, *nad4*-2, and *rps19*-p. Expression of a new chimeric ORF “*orf72”* in wild cabbage has been found to be related to the CMS phenotype [50]. This ORF contained parts of *atp9* and expressed in CMS line, but not in the euplasmic line. It was also found that expression of *orf224/atp6* chimeric gene is correlated with CMS trait in *Brassica napus*[51].

## Conclusions

Next generation sequencing technologies are powerful tools to study the heteroplasmy and substoichiometric changes of mitochondrial genomes in chondriome of plants, especially alloplasmic lines. Several heteroplasmic regions were observed within genes and also intergenic regions. The amount of structural changes in the (lo) durum lines was considerably higher than its cytoplasm donor. The rearrangements and nucleotide changes in the mitochondrial genome of the alloplasmic line that occurred in less than half a century was greater than the changes observed between mitochondrial genome of the *T. turgidum* and the *T. aestivum* which remained almost intact for 100 centuries of evolution. Evidence of paternal leakage was also observed by analyzing *nad9* and o*rf359* among all three lines. The significance of changes in the genes received from the paternal donor emphasizes that other mechanisms such as recombination, mutation, and substoichiometric shifts are active in the alloplasmic condition. Together the newly formed ORFs, differences in gene sequences and copy numbers, heteroplasmy and substoichiometric changes demonstrate the potential of the alloplasmic condition to accelerate evolution towards forming new mitochondrial genomes. Inducing changes in the chondriome and selection for the best mitotypes that fit the growing conditions would definitely broaden the paths for crop improvement in the future.

## Methods

### Plant material

Three lines from *Triticum-Aegilops* genera, *T. turgidum* ssp. *turgidum conv. durum* (Desf.) selection 56–1*, Ae. longissima* S.&M. (G759)*,* and the alloplasmic durum line [(lo) durum] were used for sequencing of the mitochondrial genomes. The (lo) durum wheat is a male sterile alloplasmic line where the cytoplasm is derived from *Ae. longissima* and the nucleus from *T. turgidum* carrying a nuclear species cytoplasm-specific gene from *T. timopheevii* (*scs*^*ti*^) that is necessary for proper compatibility [39]. This line is maintained by crossing to euplasmic durum [(d) – –] line 56–1, with no copy of the *scs* gene. Seeds produced are half plump and viable carrying the *scs*^*ti*^ locus, and half are shriveled and inviable. All the plants studied, were grown in the greenhouse and kept in dark for 2 weeks prior to mitochondrial isolation.

### Mitochondrial DNA isolation and amplification

Mitochondria isolation was conducted according to the protocol developed by Triboush et al. [35] with minor modifications. Before mitochondrial isolation leaf tissues were rinsed with 4°C distilled water and transferred to the cold room (4°C). All subsequent steps of procedure were performed in the cold room to prevent organelle degradation. Tissue homogenization was performed using the hand blender. Homogenized tissue was filtered through nylon woven mesh with 50 microns opening (Nitex). Samples were centrifuged to isolate intact mitochondria. Nuclear and chloroplast DNA contaminations were removed from the sample by DNaseI (Sigma-Aldrich Co, St. Louis, USA) treatment at 37°C for 30 minutes. Mitochondrial DNA was isolated immediately after stopping DNaseI activity using Mammalian Genomic DNA Miniprep Kit (Sigma-Aldrich Co, St. Louis, USA). To check for the proportion of probable nuclear and chloroplastic DNA contamination a quantitative real time PCR assay, using (7900HT Fast Real-Time PCR System, Applied Biosystems™) was performed. Three genome specific primers were applied for QRT-PCR reactions using TaKaRa SYBR® Premix Ex Taq™ II (Perfect Real Time, Takara Bio, Madison, USA). Gene specific primers for chloroplast (gene *psb60*), mitochondrial (gene *nad3*) and nuclear genomes (retrojunction nuclear marker) were used to test purity (Additional file 3: Table S3). No amplification of nuclear DNA was detected in any sample of mitochondrial DNA while amplification occurred using total DNA as a positive control (data not presented). Mitochondrial DNA was amplified following the manufacturer’s protocol for the Illustra™ GenomiPhi™ V2 DNA Amplification Kit (GE Healthcare, Fairfield, USA). After amplification, aliqouts of DNA were separated on 1% agarose gels for quality and quantity confirmation. Amplified DNA was acetone precipitated [52] and quantified using the Nanodrop spectrophotometer (Thermo Fisher Scientific Inc, USA). Approximately 20 μg of each mtDNA sample, dissolved in water was used for sequencing.

### Mitochondrial genome sequencing

Shot-gun, one-end sequencing by 454 Pyrosequencing™ was performed at USDA-ARS, Albany, CA, USA. An additional mtDNA concentration measurement was done using Quant-iT™ PicoGreen® dsDNA Assay Kit (Invitrogen™, USA). The mitochondrial DNA was fragmented by nebulization and fragments from 500 to 700 bp were selected for sequencing. Sequencing was done using the 454 GS FLX Titanium sequencer, with GS Titanium General Library Prep Kit, GS Titanium SV emPCR kit, GS Titanium LV emPCR kit, GS Titanium sequence kit XLR70 and GS Titanium PicoTiterPlate kit 70x75 according to manufacturer protocols.

### Mitochondrial genome assembly

Genome assembly was completed using the software MIRA3 [53] on a PC/UNIX platform. Reference assembly for each genome was performed with the *T. aestivum* (Genbank, NC_007579.1) mitochondrial genome as a backbone and *Tripsacum dactyloides* (Genbank, DQ984517) was used as negative control for assembly. The *de novo* assemblies were also performed for mitochondrial genome of each line. To assemble the (lo) durum genome, *de novo* and reference assembly were used at the same time, with the *T. aestivum* genome as reference, to partially guide the assembly within common regions of both genomes. The GAP4 software [54] was used to manually edit/join contigs obtained directly from the assembly software to create the final consensus sequences. Joining was performed only for contigs with an overlap greater than 100 bp and less than 1% dissimilarity in the overlapping region. Artemis: DNA Sequence Viewer and Annotation Tool software was used to create images of the mitochondrial genome assembly [55]. Visualization of the reference genome assembly was done using Tablet software [56]. For processing pictures IrfanView 4 (Irfan Skiljan, Wiener Neustadt, Austria) was used.

To perform *de novo* genome assembly using one end sequencing data we relied only on the nucleotide sequence of the read. Current assembly software programs were not able to complete assembly of the mitochondrial genome properly with one end data without extensive manual trimming, joining and large contig editing. Additionally due to the presence of long and short repeated sequences (present multiple times in mitochondrial genomes) and the high level of heteroplasmy, a single contig could not be created for each genome using only the algorithm implemented in the software. To overcome these difficulties, assembly was performed by extension of each contig separately against all reads to create overlaps and to find sequence spanning contigs.

### Gene finding, ORF prediction and genome annotation

Gene sequences and names were annotated based on the *T. aestivum* mitochondrial genome [35]. The NCBI database was searched for plant mitochondrial genome sequences. An open reading frame search was performed using NCBI ORF Finder according to standard translation table. The ORF’s were named arbitrarily starting from 0 for sequences being 300 bp or longer. The ORF comparison between the three mitochondrial genomes was performed and unique ORF’s were reported for each species with comparison to the other two genomes using the BLAST algorithm. Only ORFs which were 95% or less of the query size were considered as unique and these ORFs were reported and described.

### Mitochondrial genome polymorphism

The assembled sequences for each genome were used as references for SNP detection. The raw reads from the same line were mapped against the reference sequence for that line. The raw assembly files from GAP4 software [54] were used to search for polymorphism for each sequenced position. Only regions of the genome with at least a 10x threshold of genome coverage were used for SNP search. A SNP was reported only when the number of different nucleotides in the same position was observed in more than 10% of the reads and sequence coverage of that region was at least 10X. With this criterion only 0.3% (1,283 bp) to 1% (3,962 bp) of the genome were not considered in the analysis (Additional file 7: Table S2). A SNP search between mitochondrial genomes was performed by using the BLAST search due to the high number of rearrangement between genomes. An identity of 95% was used as the cut off for finding similar regions through BLAST search, and in that region SNPs were detected as described before. An error rate (false positive) for the SNP search was established comparing 17,046 bp of gene space (which are: *atp1, atp9, rps13, cox3, matR, rps1, ccmFN, cob, rpl16, rps3, ccmFC, orf359, nad4, orf240, ccmB, atp4, nad2, orf173, nad9 orf349, rps2*) between sequenced species and the *T. aestivum* reference. Only nucleotide changes were considered in the SNP analysis. Nucleotide insertion/deletion events were not reported.

### Confirmation of sequence assemblies and *atp6*gene structure

The sequence of the *atp6-1* gene in all three sequenced species was confirmed by Sanger sequencing of the complete gene, using the mtDNA and total genomic DNA. Additionally, the presence/absence and sequence of *atp*6-1 was confirmed by PCR using atp6LO, atp6TU as forward primers in combination with a common reverse primer (atp6TU-R) designed in the core region of the gene (Additional file 3: Table S3). PCR with specific primers was performed to confirm proper contig structure and structural differences between the *T. turgidum* and the (lo) durum genomes. The primer design was made using OLIGO Primer Analysis Software v.7 [57] following by Primer-BLAST [58].

Final genome assembly was used to explain the overall structural differences between the genomes. Assignment of gene positions in each genome, allowed the creation of gene synteny maps showing only gene relationships between mitochondrial genomes in sequenced species. Double ACT v2 (Health Protection Agency) together with ACT software (Artemis Comparison Tool) from Sanger Institute was used to demonstrate syntenic differences and gene positions across sequenced mitochondrial genomes.

## Electronic supplementary material

Additional file 1: Table S1: Groups of genes present in the mitochondrial genomes of *Triticum turgidum*, (lo) durum and *Aegilops longissima* without polymorphism. (DOCX 15 KB)

Additional file 2: Figure S1: The sequence of *atp6* gene including the conserved regions of mitochondrial genome surrounding both alleles. Region in blue represents a pre-sequence of the *atp6* gene, starting from ATG codon. Region in green represent the conserved region and gray bars shows polymorphism found within core region of the gene. (DOCX 266 KB)

Additional file 3: Table S3: The PCR primers for DNA quantification and atp6 sequence confirmation. These primers were used to establish level of chloroplast and nuclear DNA contamination in the mitochondrial DNA samples and to differentiate between alleles of the *atp6* gene. (DOCX 16 KB)

Additional file 4: Figure S2: The *nad9* nucleotide sequence comparison between (lo) durum and the parental lines. Three SNP’s were recognized (light gray boxes) in comparison to the *Triticum turgidum*, one of them (dark gray box) was found only in the (lo) durum line. The four-nucleotide deletion in the *Ae. longissimum* creates a STOP codon at the base 157. An additional di-nucleotide change (CA/TG^134-135^) in the (lo) durum is indicated by the orange box. (DOCX 170 KB)

Additional file 5: Figure S3: The *nad6* nucleotide sequence comparison between the (lo) durum and the parental lines. Three SNPs were recognized and two di-nucleotide changes (light gray boxes) in comparison to the *T. turgidum*. The highly polymorphic region starts at position 703 as indicated by the arrow. (DOCX 156 KB)

Additional file 6: Figure S4: The *rps19-p* nucleotide sequence comparison between the (lo) durum and the parental lines. In the *T. turgidum* there is a nine nucleotide deletion in *rps19-p* when compared to the (lo) durum and the *Ae. longissimum* which share the same allele. (DOCX 97 KB)

Additional file 7: Table S2: Number of heteroplasmic single nucleotide polymorphisms (HSNPs) and clusters of polymorphism found within each of the three sequenced mitochondrial genomes. (DOCX 16 KB)

Additional file 8: Figure S5: The *orf359* nucleotide sequence comparison between the (lo) durum and the parental lines*.* The sequence of *orf359* found in the alloplasmic line was highly polymorphic when compared to the *T. turgidum* gene*.* We identified 48 SNP’s, three di-nucleotide changes, one tri-nucleotide change, and one three nucleotide insertion. The *Ae. longissima* sequence assembly does not have a copy of *orf359*. (DOCX 164 KB)

Additional file 9: Figure S6: Structure of *orf113* specific to the alloplasmic durum line. Database search showed similarity to *rps2*, *cox1*, *nad4-2*, *rps19-p* mitochondrial genes. Shown are the relative fragment size and location of each gene found in the ORF. (DOCX 27 KB)
