# Supplementary material for: Accelerated evolution of the mitochondrial genome in an alloplasmic line of durum wheat
Source: BMC Genomics. 2014 Jan 25;15(1):67. doi: 10.1186/1471-2164-15-67 (PMC3942274; doi:10.1186/1471-2164-15-67)
Supplement: Supplementary file 1 — Additional file 1: Table S1: Groups of genes present in the mitochondrial genomes of Triticum turgidum, (lo) durum and Aegilops longissima without polymorphism. (DOCX 15 KB) [file 12864_2013_7007_MOESM1_ESM.docx]

Table S1. Groups of genes present in mitochondrial genomes of *Triticum turgidum*, (lo) durum and *Aegilops longissima* without polymorphism.

| **Complex I** |
| --- |
| *nad1-1, nad1-2, nad1-3, nad1-4, nad1-5* |
| *nad2-1, nad2-2, nad2-3, nad2-4, nad2-5* |
| *nad4-1, nad4-2, nad4-3, nad4-4* |
| *nad4L* |
| *nad5-1, nad5-2, nad5-3, nad5-4* |
| *nad7-1, nad7-2, nad7-3, nad7-4, nad7-5* |
| **Complex IV** |
| *cox1,cox2-2* |
| **Complex V** |
| *apt4, atp9* |
| **Complex c biogenesis** |
| *ccmB, ccmFC* |
| **Other proteins** |
| *matR* |
| **Ribosomal proteins** |
| *rps3-1, rps3-2, rps3-3, rps3-4* |
| *rps7,rps12, rpl16* |
| **tRNA** |
| *Asn tRNA, Asp tRNA, Cys tRNA, Gln-1 tRNA, Gln-II tRNA, Gln-III tRNA*, Glu tRNA, Lys-1 tRNA, Lys-2 tRNA, Lys-3 tRNA*, Met-1 tRNA, Met-2 tRNA, Met -3 tRNA, Met- IV tRNA, Phe tRNA, Pro -1 tRNA, Pro - 2 tRNA, Ser -1 tRNA, Ser -2 tRNA, Ser -3 tRNA, Trp tRNA, Tyr tRNA* |
| **rRNA** |
| *rrn5-1, rrn5-2^#^, rrn5-3^#^, rrn18-1, rrn18-2^#^, rrn18-3^#^ , rrn26-1, rrn26-2, rrn26-3^$^* |

* Designate copies of genes not present in the final genome assembly, but present in multiple copies in raw assembly data.

^#^ Copies of genes not present in final assembly in *Aegilops longissimum* and *Triticum Tauschii.*

^$^ Only part of the gene was found in the final assembly of (lo) durum.

Arabic and roman numerals designate alleles of the same genes.
