# Supplementary material for: Accelerated evolution of the mitochondrial genome in an alloplasmic line of durum wheat
Source: BMC Genomics. 2014 Jan 25;15(1):67. doi: 10.1186/1471-2164-15-67 (PMC3942274; doi:10.1186/1471-2164-15-67)
Supplement: Supplementary file 2 — Additional file 2: Figure S1: The sequence of atp6 gene including the conserved regions of mitochondrial genome surrounding both alleles. Region in blue represents a pre-sequence of the atp6 gene, starting from ATG codon. Region in green represent the conserved region and gray bars shows polymorphism found within core region of the gene. (DOCX 266 KB) [file 12864_2013_7007_MOESM2_ESM.docx]

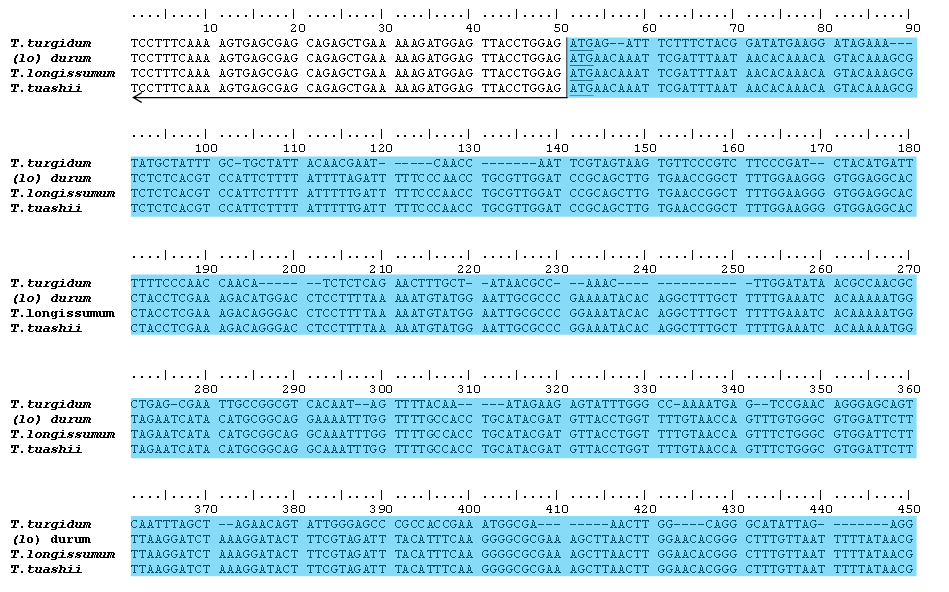


**A**

*T. turgidum*

(lo) durum

*Ae. longissima*

*T. turgidum*

(lo) durum

*Ae. longissima*

*T. turgidum*

(lo) durum

*Ae. longissima*

*T. turgidum*

(lo) durum

*Ae. longissima*

*Triticum turgidum*

(lo) durum

*Aegilops longissima*


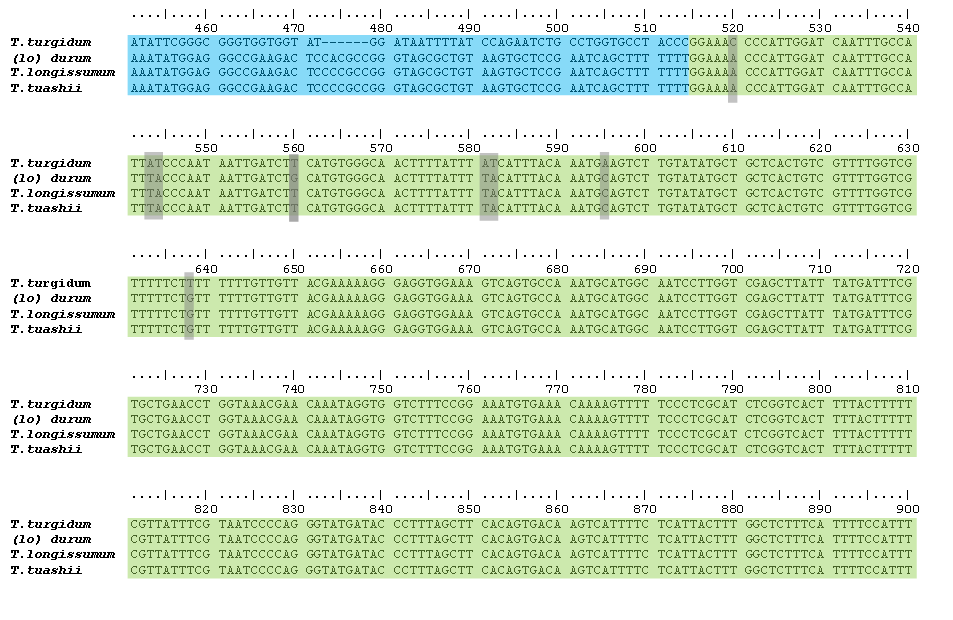


**B**

*T. turgidum*

(lo) durum

*Ae. longissima*

*T. turgidum*

(lo) durum

*Ae. longissima*

*T. turgidum*

(lo) durum

*Ae. longissima*

*T. turgidum*

(lo) durum

*Ae. longissima*

*T. turgidum*

(lo) durum

*Ae. longissima*

*T. turgidum*

(lo) durum

*Ae. longissima*

*T. turgidum*

(lo) durum

*Ae. longissima*

*T. turgidum*

(lo) durum

*Ae. longissima*

*T. turgidum*

(lo) durum

*Ae. longissima*

*T. turgidum*

(lo) durum

*Ae. longissima*

**Figure S1.** The sequence of *atp6* gene sequence including conserved regions of mitochondrial genome surrounding both alleles of the gene. Region in blue represents a pre-sequence of the *atp6* gene, starting from ATG codon. Region in green represent the conserved region and gray bars shows polymorphism found within core region of the gene.


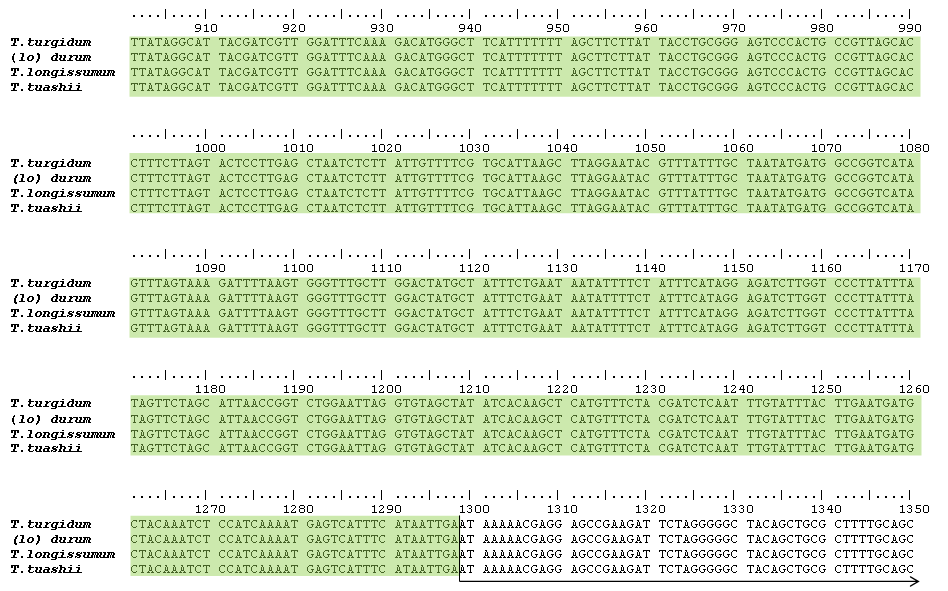


**C**
