# Supplementary material for: Accelerated evolution of the mitochondrial genome in an alloplasmic line of durum wheat
Source: BMC Genomics. 2014 Jan 25;15(1):67. doi: 10.1186/1471-2164-15-67 (PMC3942274; doi:10.1186/1471-2164-15-67)
Supplement: Supplementary file 3 — Additional file 3: Table S3: The PCR primers for DNA quantification and atp6 sequence confirmation. These primers were used to establish level of chloroplast and nuclear DNA contamination in the mitochondrial DNA samples and to differentiate between alleles of the atp6 gene. (DOCX 16 KB) [file 12864_2013_7007_MOESM3_ESM.docx]

Table S3. The PCR primers for DNA quantification, atp6 sequence confirmation. These primers were used to establish level of chloroplast and nuclear DNA contamination in the mitochondrial DNA samples and to differentiate between alleles of atp6 gene.

| **Genome (gene)** | **Primer name** | **Sequence 5' - 3'** | **Amplicon size (bp)** | **Primer source** |
| --- | --- | --- | --- | --- |
| Chloroplast (*psb60*) | psbB-60F | ATGGGTTTGCCTTGGTATCGTGTTCATAC | 355 | Heinze B. 2007 |
|  | psbB-61R | TCCCAATAYACCCAATGCCAGATAG |  |  |
| Mitochondria (*nad3*) | nad3-F | GCACCCCTTTTCCATTCATA | 357 | This paper |
|  | nad3-R | TCGGAATTTGCACCTATTTG |  |  |
| Nucleus (retro-junction) | cfp1589 | GTTTCGTTCATGCGATATGTGCC | 321 | Paux E. at al. 2008 |
|  | cfp1589 | ATCTCTACCCCCCGCCCC |  |  |
| *atp6* | atp6LO-F | TCCGCAGCTTGTGAACCGGC | 843 | This paper |
|  | atp6LO-R | GCAGTGGGACTCCCGCAGGTA |  |  |
|  | atp6TU-F | AACGCCAACGCCTGAGCGAA | 642 |  |
|  | atp6TU-R | GCAGTGGGACTCCCGCAGGTA |  |  |
