# Supplementary material for: Accelerated evolution of the mitochondrial genome in an alloplasmic line of durum wheat
Source: BMC Genomics. 2014 Jan 25;15(1):67. doi: 10.1186/1471-2164-15-67 (PMC3942274; doi:10.1186/1471-2164-15-67)
Supplement: Supplementary file 4 — Additional file 4: Figure S2: The nad9 nucleotide sequence comparison between (lo) durum and the parental lines. Three SNP’s were recognized (light gray boxes) in comparison to the Triticum turgidum, one of them (dark gray box) was found only in the (lo) durum line. The four-nucleotide deletion in the Ae. longissimum creates a STOP codon at the base 157. An additional di-nucleotide change (CA/TG134-135) in the (lo) durum is indicated by the orange box. (DOCX 170 KB) [file 12864_2013_7007_MOESM4_ESM.docx]

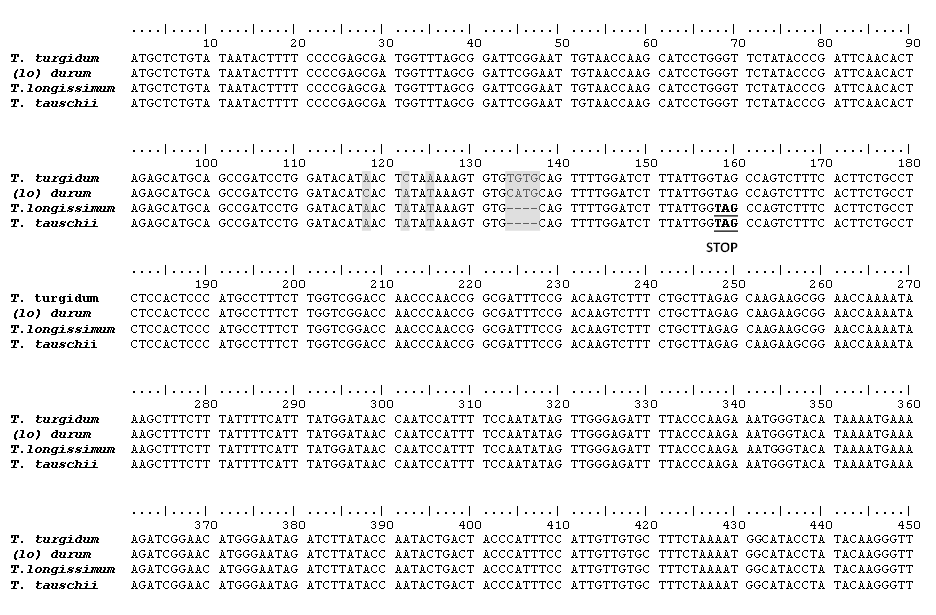


*Triticum turgidum*

(lo) durum

*Aegilops longissima*

*T. turgidum*

(lo) durum

*Ae. longissima*

*T. turgidum*

(lo) durum

*Ae. longissima*

*T. turgidum*

(lo) durum

*Ae. longissima*

*T. turgidum*

(lo) durum

*Ae. longissima*

**A**

**Figure S2.** The *nad9* nucleotide sequence comparison between (lo) durum and the parental lines . Three SNP’s were recognized (light gray boxes) in comparison to *Triticum turgidum*, one of them (dark gray box) was found only in (lo) durum line. The four-nucleotide deletion in *T. longissimum* creates a STOP codon at the base 157. An additional di-nucleotide change (CA/TG^134-135^) in (lo) durum is indicated by the (orange box).


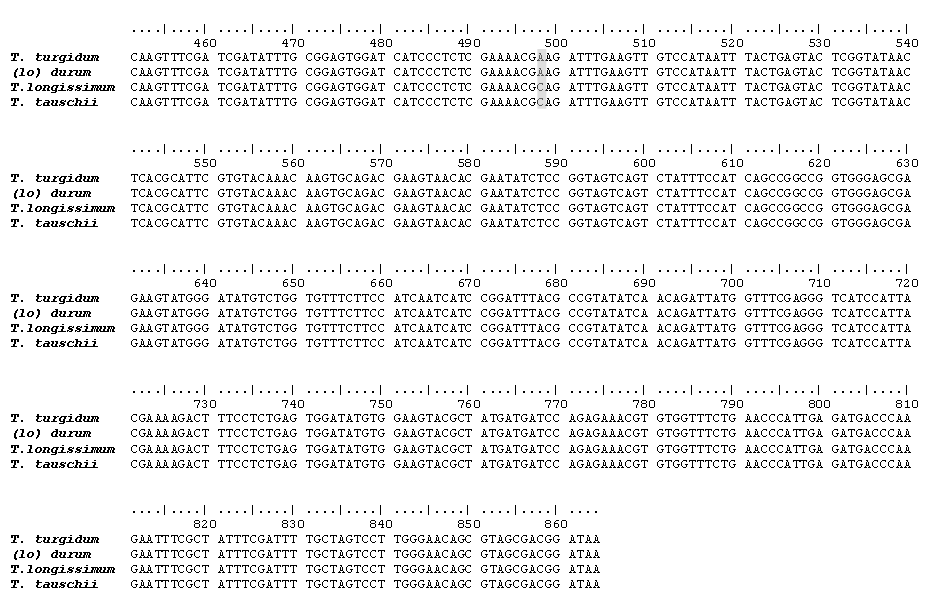


*T. turgidum*

(lo) durum

*Ae. longissima*

*T. turgidum*

(lo) durum

*Ae. longissima*

*T. turgidum*

(lo) durum

*Ae. longissima*

*T. turgidum*

(lo) durum

*Ae. longissima*

*T. turgidum*

(lo) durum

*Ae. longissima*

**B**
