# Supplementary material for: Accelerated evolution of the mitochondrial genome in an alloplasmic line of durum wheat
Source: BMC Genomics. 2014 Jan 25;15(1):67. doi: 10.1186/1471-2164-15-67 (PMC3942274; doi:10.1186/1471-2164-15-67)
Supplement: Supplementary file 5 — Additional file 5: Figure S3: The nad6 nucleotide sequence comparison between the (lo) durum and the parental lines. Three SNPs were recognized and two di-nucleotide changes (light gray boxes) in comparison to the T. turgidum. The highly polymorphic region starts at position 703 as indicated by the arrow. (DOCX 156 KB) [file 12864_2013_7007_MOESM5_ESM.docx]

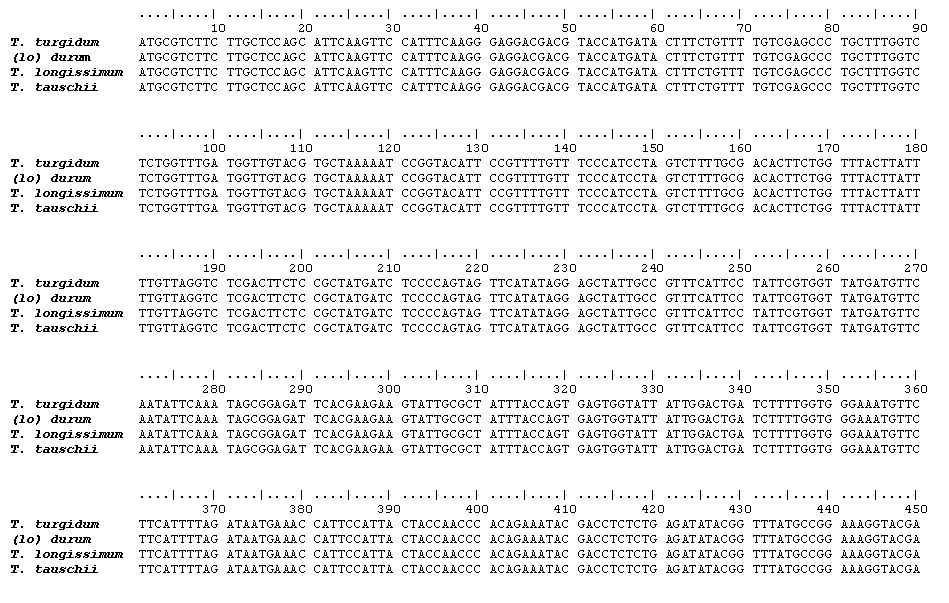


**A**

*T. turgidum*

(lo) durum

*Ae. longissima*

*T. turgidum*

(lo) durum

*Ae. longissima*

*T. turgidum*

(lo) durum

*Ae. longissima*

*T. turgidum*

(lo) durum

*Ae. longissima*

*Triticum turgidum*

(lo) durum

*Aegilops longissima*

**Figure S3.** The *nad6* nucleotide sequence comparison between (lo) durum and the parental lines. Three SNPs were recognized and two di-nucleotide changes (light gray boxes) in comparison to *Triticum turgidum*. The highly polymorphic region starts at position 703 as indicated by the arrow.


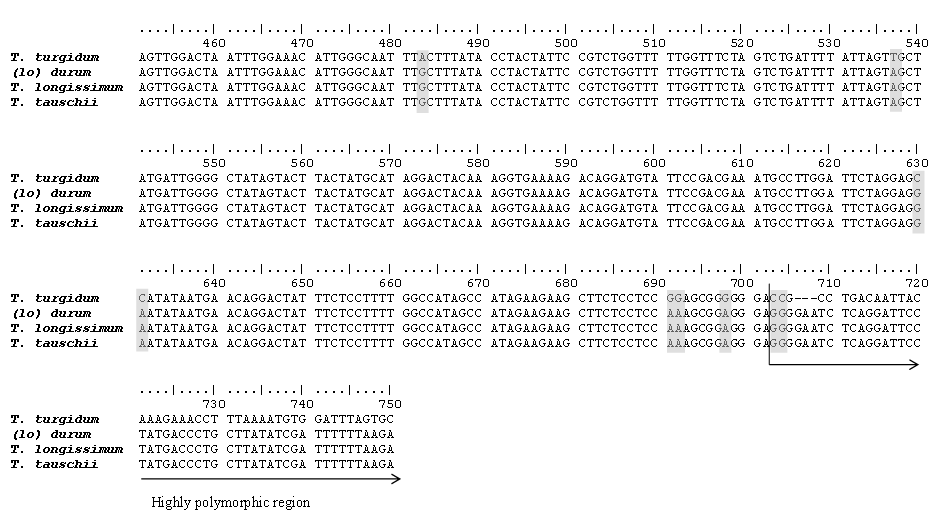


*T. turgidum*

(lo) durum

*Ae. longissima*

*T. turgidum*

(lo) durum

*Ae. longissima*

*T. turgidum*

(lo) durum

*Ae. longissima*

*T. turgidum*

(lo) durum

*Ae. longissima*
