# Supplementary material for: Accelerated evolution of the mitochondrial genome in an alloplasmic line of durum wheat
Source: BMC Genomics. 2014 Jan 25;15(1):67. doi: 10.1186/1471-2164-15-67 (PMC3942274; doi:10.1186/1471-2164-15-67)
Supplement: Supplementary file 6 — Additional file 6: Figure S4: The rps19-p nucleotide sequence comparison between the (lo) durum and the parental lines. In the T. turgidum there is a nine nucleotide deletion in rps19-p when compared to the (lo) durum and the Ae. longissimum which share the same allele. (DOCX 97 KB) [file 12864_2013_7007_MOESM6_ESM.docx]

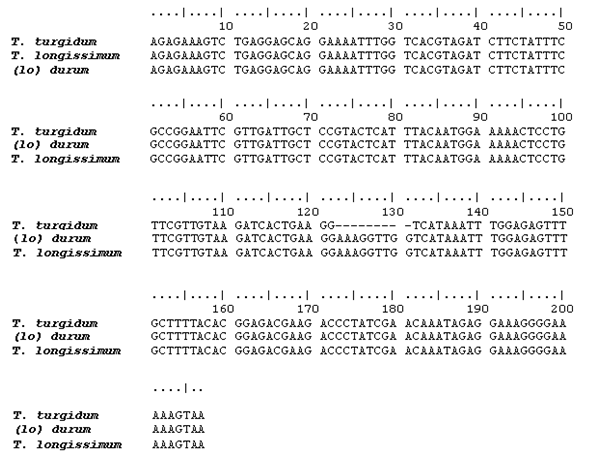


**Figure S4.** The  *rps19-p* nucleotide sequence comparison between (lo) durum and the parental lines. In *Triticum turgidum* there is a nine nucleotide deletion in *rps19-p* when compared to (lo) durum and *Ae. longissimum* which share the same allele.

*T. turgidum*

*Ae. longissima*

(lo) durum

*T. turgidum*

*Ae. longissima*

(lo) durum

*T. turgidum*

*Ae. longissima*

(lo) durum

*T. turgidum*

*Ae. longissima*

(lo) durum

*Triticum turgidum*

*Aegilops longissima*

(lo) durum
