# Supplementary material for: Accelerated evolution of the mitochondrial genome in an alloplasmic line of durum wheat
Source: BMC Genomics. 2014 Jan 25;15(1):67. doi: 10.1186/1471-2164-15-67 (PMC3942274; doi:10.1186/1471-2164-15-67)
Supplement: Supplementary file 7 — Additional file 7: Table S2: Number of heteroplasmic single nucleotide polymorphisms (HSNPs) and clusters of polymorphism found within each of the three sequenced mitochondrial genomes. (DOCX 16 KB) [file 12864_2013_7007_MOESM7_ESM.docx]

| **Species** | **SNP*** | **HSNP density (Number/bp)** | **HSNP Blocks^$^** | **1-10x^#^** |
| --- | --- | --- | --- | --- |
| Triticum turgidum | 93 | 1/4859 | 22 (151) | 0.3% (1283bp) |
| (lo) durum | 61 | 1/7091 | 15 (155) | 0.9% (3903bp) |
| Aegilops longissima | 98 | 1/6542 | 27 (246) | 1.0% (3962bp) |

**Table S2.** Number of heteroplasmic single nucleotide polymorphisms (HSNPs) and clusters of polymorphism found within each of the three sequenced mitochondrial genomes.

*****HSNP is heteroplasmic single nucleotide polymorphism found in a particular position, only nucleotide changes were considered (insertion/deletions were excluded from analysis).

^$^HSNP blocks describes genomic regions with an increased density of HSNPs in close proximity (with less than 15 bp between HSNPs).

^#^Percent of regions with coverage of 1-10x in the assembled genomes.
