# Supplementary material for: Accelerated evolution of the mitochondrial genome in an alloplasmic line of durum wheat
Source: BMC Genomics. 2014 Jan 25;15(1):67. doi: 10.1186/1471-2164-15-67 (PMC3942274; doi:10.1186/1471-2164-15-67)

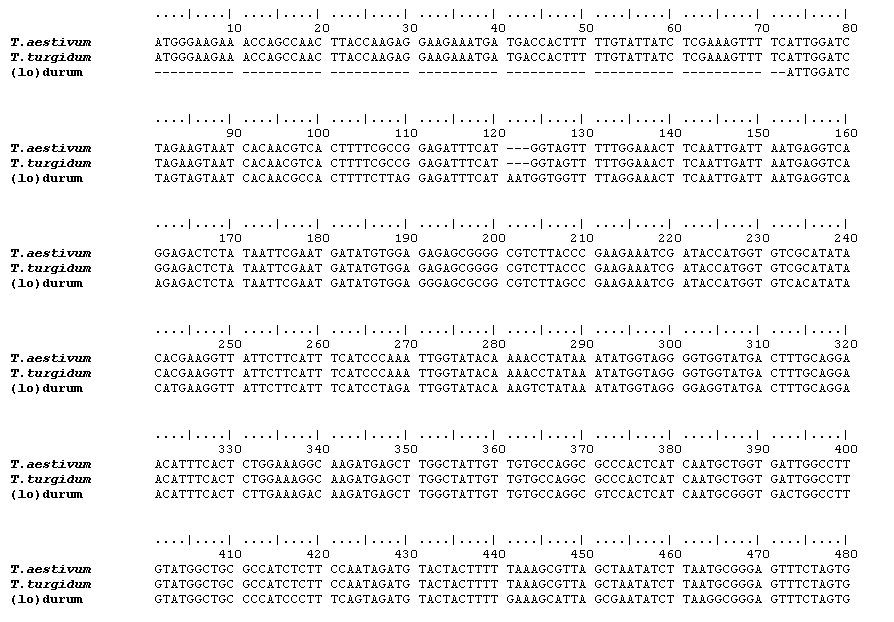


**A**


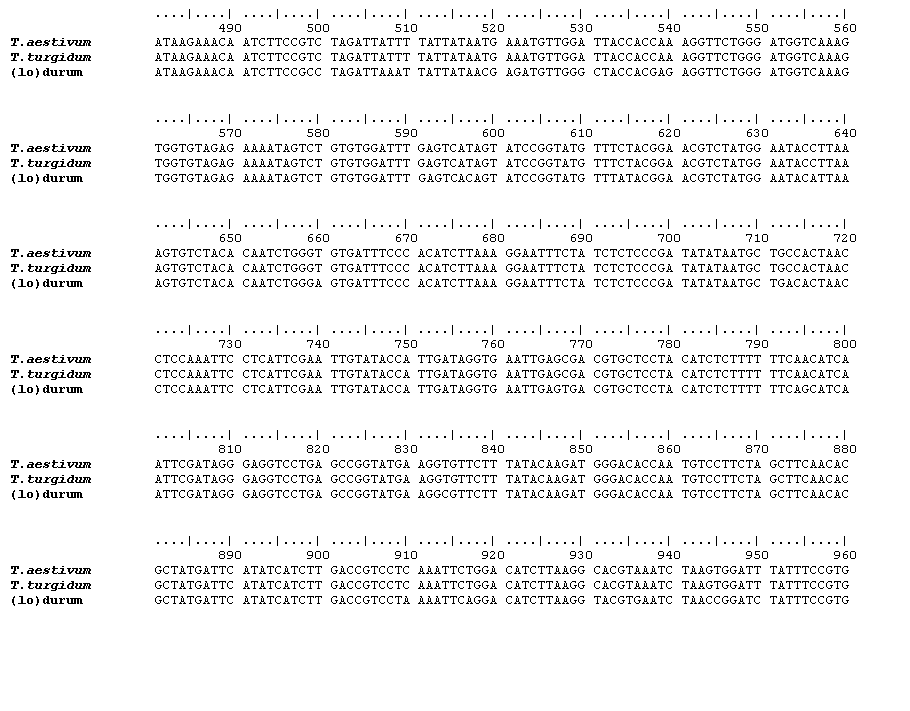


**B**

**Figure S5.** The *orf359* nucleotide sequence comparison between (lo) durum and the parental lines*.* The sequence of *orf359* found in the alloplasmic line was highly polymorphic when compared to the *Triticum turgidum* gene*.* We identified 48 SNP’s, three di-nucleotide changes, one tri-nucleotide change, and one three nucleotide insertion. The *Ae. longissima* sequence assembly does not have a copy of *orf359*.

**C**


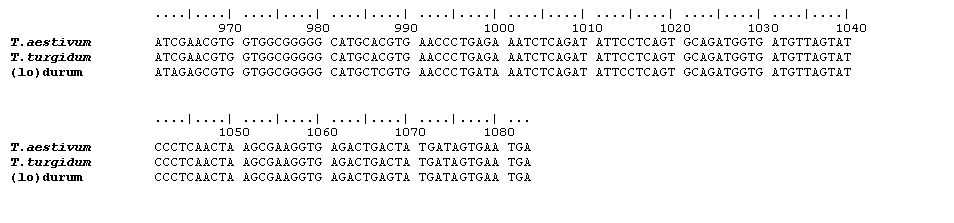

Supplement: Supplementary file 8 — Additional file 8: Figure S5: The orf359 nucleotide sequence comparison between the (lo) durum and the parental lines. The sequence of orf359 found in the alloplasmic line was highly polymorphic when compared to the T. turgidum gene. We identified 48 SNP’s, three di-nucleotide changes, one tri-nucleotide change, and one three nucleotide insertion. The Ae. longissima sequence assembly does not have a copy of orf359. (DOCX 164 KB) [file 12864_2013_7007_MOESM8_ESM.docx]
