# Supplementary material for: Accelerated evolution of the mitochondrial genome in an alloplasmic line of durum wheat
Source: BMC Genomics. 2014 Jan 25;15(1):67. doi: 10.1186/1471-2164-15-67 (PMC3942274; doi:10.1186/1471-2164-15-67)
Supplement: Supplementary file 9 — Additional file 9: Figure S6: Structure of orf113 specific to the alloplasmic durum line. Database search showed similarity to rps2, cox1, nad4-2, rps19-p mitochondrial genes. Shown are the relative fragment size and location of each gene found in the ORF. (DOCX 27 KB) [file 12864_2013_7007_MOESM9_ESM.docx]

STOP

codon

START

codon

*rps2*

*cox1*

*nad4-2*

*rps19-p*

**Figure S6**. Structure of *orf113* specific to the alloplasmic durum line. Database search showed similarity to *rps2*, *cox1*, *nad4-2*, *rps19-p* mitochondrial genes. Shown are the relative fragment size and location of each gene found in the ORF.
